# Predictors of academic performance with due focus on undernutrition among students attending primary schools of Hawa Gelan district, Southwest Ethiopia: a school based cross sectional study

**DOI:** 10.1186/s40795-017-0138-2

**Published:** 2017-03-27

**Authors:** Frehiwot Abebe, Ayele Geleto, Lelisa Sena, Cherinet Hailu

**Affiliations:** 1 Oromia Regional Health Bureau, Nutrition directorate, Addis Ababa, Oromia Region Ethiopia; 2 School of Public Health, College of Health and Medical Sciences, Haramaya University, Harar, Ethiopia; 3 Department of Public Health, School of Public Health, College of Medicine and Health Sciences, Jimma University, Jimma, Ethiopia

**Keywords:** Undernutrition, Academic performance, Primary schools, Students

## Abstract

**Background:**

More than a quarter of children living in Sub-Saharan Africa are underweight. Nutritional deficiency in children increases the risk of infection and affects their mental development. However, there was scarcity of research findings that clearly indicate determinants of academic performance. Therefore, this study was aimed at determining predictors of academic performance with due focus on undernutrition among students attending primary schools.

**Methods:**

School based cross sectional study was conducted in February, 2016 among 630 randomly selected students attending primary schools in Hawa Galan woreda. Data were collected through parents/guardians interview, anthropometric measurement of children and school record review. Data were entered into Epidata version 3.1 and analyzed with SPSS Version 20. Anthropometric data were analyzed by WHO Anthro plus software. Pearson’s correlation analysis was performed to determine correlation between academic performance and undernutrition. Logistic regression analysis was also performed to assess predictors of academic performance and *p* < 0.05 was used to declare significant association.

**Result:**

Prevalence of stunting, wasting and underweight in this study were 20.6%, 12.7% and 14.3% respectively. This study found a significant correlation between underweight (*r* = 0.222, *P* = 0.040), stunting (*r* = 0.214, *P* = 0.034) and academic performance. Multiple logistic regression analysis also indicated that being female [AOR 1.48; 95% CI (1.16, 3.82)], attending above grade 4 [AOR 2.12; 95% CI (1.98, 4.87)], having educated parents [AOR 2.18; 95% CI (1.43, 4.72)], coming from households with monthly income of more than 2000ETB (~USD91) [AOR 2.85; 95% CI (2.01, 5.21)] and having no parental support during homework [AOR 0.57; 95% CI (0.19, 0.98)] were significantly associated with students’ academic performance.

**Conclusion:**

Stunting and underweight were found to be correlated with academic performance of students attending primary schools. Nutritional interventions should be considered in the study area. Parents should be encouraged to be involved in their children’s schooling.

## Background

Schooling is an instrument of individual and social change, increasing the probability of general well-being. A decade after adoption of the Education for All (EFA) goals in 2000, many children are still out of school [1]. For millions of children, hunger is one of the obstacles to school participation. Primary education is a critical stage in the development of the consciousness and personality of the children. At this stage children are extremely inquisitive and elementary education must encourage this tendency among children. Nutrition is an endogenous factor that affects the learning ability and skills of children at school. The relationship between nutrition and school performance of school-age population in developing countries has been of interest to many researchers due to the frequent observation of poor school performance among malnourished students [2].

More than a quarter of children below fifteen years of age in Sub-Saharan Africa are underweight due to malnutrition. Nutritional deficiencies in children increase the risk of infection and affect their mental development [3]. Although Ethiopia has already achieved a remarkable progress in reducing under-five mortality in the last decades, under nutrition among children is still a common health problem [4]. In Ethiopia, food insecurity occurs widely and acute malnutrition is growing above the global thresholds that define an emergency [5]. Children who do not consume adequate amounts of key nutrients were unable to work to their full potential at school. Several studies revealed that low birth weight is associated with grade repetition or poor school performance [6–9].

Cognitive development and brain physiology among children and adolescents requires access to sufficient and nutrient rich food at early stages of life. Recurrent food shortage as experienced in Ethiopia [10] may result in undernutrition which results in developmental impairments including poor learning capacity in children [11, 12]. Malnutrition adversely affects school attendance, and academic performance and social skills among children and adolescents [13, 14]. Stunting which reflects a chronic nutritional deprivation adversely affect the cognitive development of the children. A stunted child is not only unable to enroll in school at the right age but also cannot attend classes properly and lead to high student drop-outs rate [2]

A robust literature links brain function outcomes with nutritional status. Research findings indicate that the brain needs to be seen as being affected by nutrition, the concentration of amino acids and choline which let the brain create and use many of its neurotransmitters [15]. All substances found in food are important to brain development and function. Proteins found in foods are used to make brain tissue. A lack of protein, also known as Protein Energy Malnutrition, led to poor school performance among children and lead to young children to be passive which affects their social and emotional development [16].

Even though there was high prevalence of malnutrition in Ethiopia, its association with educational performance among students attending primary schools was rarely assessed. However, having clear information on the nutritional status and its effect on academic achievement of students in general and among students attending primary schools in particular helps to take corrective action in order to solve the problem. Therefore, this study was aimed at assessing predictors of academic performance with due focus on undernutrition among students attending primary schools at Hawa Gelan district, Kellem Wollega, south west Ethiopia. The study result provided general information for decision makers, health service and educational managers and other development partners involved in promotion of health and education.

## Methods

### Study design and setting

A school based cross-sectional study was conducted in Hawa Galan District of Kellem Wollega Zone, Oromia Regional state, south west Ethiopia in February 2016. Hawa Gelan is one of the districts in the Kelem Welega zone of Oromia Regional state. Rob Gebeya is the administrative town of the district. According to the projection from the 2007 census the wereda’s total population for the year 2016 is estimated to be 122, 686, of whom 60,265 were male and 62,421 were female. The district has 30 rural kebeles (the smallest administrative structure) and two urban kebeles. There are six health centers and 30 functional health posts in the district. The district has 21 primary schools and four high schools. Hawa Gelan district is one of the food insecure districts in Oromia region. Up to 30 undernutrition cases are reported weekly from all health facilities of the district. [17].

### Participants of the study

All randomly selected students attending primary schools in the district paired with their parents were participated in to this study. The parents accompany their children to provide socio demographic data and parental involvement in their children schooling. However, children with edema and body swellings were excluded from the study as it can affect their weight. Students attending grade one were also excluded from the study because we used the average annual score of two consecutive semesters to determine academic performance (for grade one students this could not be obtained).

### Sample size calculation and sampling technique

Sample size was calculated using single population proportion formula. The result of previous study of 30.7% stunting among primary school in Ethiopia [17], 95% confidence interval (CI) and 5% margin of error were used to calculate the sample size. Therefore, sample was calculated as: *n =* [(1.96) ^2^x 0.307 (1–0.307)]/(0.05) ^2^ = 327. Design effect of 2 was used and the final sample size was calculated to be 654.

A multi stage sampling technique was employed to select the study participants. There were 21 primary schools in the district. These primary schools were initially stratified in to urban (fifteen) and rural (six). Then seven primary schools (two from urban and five from rural) were proportionally selected using simple random sampling (lottery) method. Then the sample size was proportionally allocated for the seven randomly selected schools taking the grades attended in to consideration (124 from Akako, 103 from Lalistu Sombo, 94 from Boni, 89 from Gecho, 84 from Haro Mechara, 82 from Hawa Fincho and 78 from Hawa Babo). Finally, students in each class were alphabetically listed and participants of the study were proportionally selected from the lists of the students in each class using systematic sampling technique.

### Data collection

Socio demographic data and information about parental involvement in their children’s schooling were collected by face to face parental/guardian interview using structured questionnaire. Anthropometric data for the students were gathered through anthropometric measurement. The questionnaire used for interview was prepared in English language after different relevant literature review and translated into *Afaan Oromo,* a local language, and then re-retranslated to English for analysis to maintain its coherence. Four clinical nurses who can speak local language were recruited as data collectors. Two BSc nurses were hired from the district to supervise the data collection process. To ensure data quality, data collectors and supervisors were given training before commencement of data collection. During the training, the objective of the study, data collection procedures and techniques including how to conduct anthropometric measurements were discussed in detail. During the training, practical exercise of data collection was done through peer interviewer and anthropometric measurement was conducted on students of the same age at a nearby school that was not selected for the real study.

### Anthropometric measurements

Anthropometric measurements were performed to obtain height and weight of the students. Weight was measured using seca digital weigh scale to the nearest 0.1 kg while the students were dressed their school uniform and without their shoes. Calibration for the weight scale was done upon every case examination. Measurement of height was obtained in a standing position to the nearest1cm using a wooden height board. All measurements were taken twice and the mean value was used for data analysis. Age of the students was collected from student’s records in case there was no confirmed age record.

Weight-for age Z-scores (WAZ) and height-for-age Z-scores (HAZ) were generated by WHO AnthroPlus Version 3.2.2 from anthropometric measures of weight, height and age; taking sex into consideration. Nutritional status of the children was determined in reference to the age and sex-specific growth charts. HAZ below -2SD of the reference population indicates stunting. WHZ below -2SD of the reference population indicates wasting. WAZ below -2SD of the reference population is underweight [17].

### Measurement of academic achievements

The overall subjects the students were given in the academic year 2015/16 were considered to determine the academic achievements of the students. Annual average score was calculated by taking the result of two consecutive semesters of the year. To examine the effect of nutritional status on educational performance, average marks of the overall subjects the students received were divided into two categories, poor score and good score, based on a cut-off mark of 50%. This cut off point was determined by considering the pass mark set by federal ministry of education, Ethiopian.

### Data analysis

Data entry was performed using EpiData version 3.1 and exported to SPSS version 20 statistical package for analysis. We employed three data analysis stages. First of all, descriptive statistics including means, ratios, standard deviations, frequency tables and percentages were used to present the results. Secondly, Pearson’s correlation test was performed to assess the linear relationship between undernutrition and the students’ academic performance. Lastly, logistic regression analysis was conducted to estimate predictors of academic achievement of students. Multivariate logistic regression analysis with crude odds ratio at 95% CI was used to determine presence of association between independent and dependent variables. The degree of association between variables was measured using adjusted odds ratio with 95% confidence interval and association was declared significant at *P* value ≤ 0.05.

### Study variables

The dependent variable in this study was academic performance of students. The independent variables for this study include participants’ nutritional status (weight for age and height for age); socio-demographic variables (sex, age, ethnicity, religion); family characteristics (marital status of the family, family support for the students, educational status of the family, monthly income of family, family size, occupation of family).

### Data quality control

The questionnaire was pretested on 32 (5%) of the sample size of similar population in non-selected school in the district. Data collectors and supervisors were selected based on their experience in the field of data collection and supervision. Field staffs were given training before the commencement of data collection. During training, the objective of the study, procedures of data collection and supervision were discussed in detail. Furthermore, each question included in the questionnaire was discussed deeply and any ambiguity was made clear. Each day, the collected data were checked for its completeness and consistence by supervisors and investigators. Data were also cleaned and rechecked after double data entry was performed.

### Ethical considerations

To conduct this study, ethical approval was obtained from Institutional Review Board (IRB) of school of public health, Jimma University. Official letters of cooperation were written to each selected schools from Jimma University. Parents/guardians of the participating students were informed about the objective of the study, risks and benefit, confidentiality of data and privacy of the information. Parents/Guardians were asked for their willingness to participate in the study and they were told that they have the right to refuse to participate at all or can interrupt the question at any time if they feel discomfort to respond for the question. Written and signed informed consent to participate in this study was obtained from the parents/guardians since our study population was children younger than 16 years. Confidentiality issue was insured by using codes and students’ identifiers were not recorded.

## Result

### Socio-demographic characteristics of the respondents

Six hundred thirty students paired with their parents were enrolled in to this study making response rate of 96.3%. The mean age of the students was 12.2 years (SD ± 2 years). Nearly equal male 305 (48.4%) and female 325 (51.6%) were participated in to the study. Majority of the study participants 409 (64.9%) were Oromo by ethnicity, and nearly two fifth, 261 (41.4%) were protestant by religion followed by Orthodox Christians 251 (39.8%). Two hundred twenty two (35.2%) and 408 (64.8%) of the respondents were attending first cycle primary school (Grade 2–4) and second cycle primary school (Grade5-8) respectively. (Table 1)

**Table 1:** Socio-demographic characteristics and parental involvement in schooling at primary schools of Hawa Gelan District, Oromia region, South west Ethiopia, 2016, (*N* = 630)

Variables	Categories	Frequency	Percentage
Age in years	<10 years	133	21.1
	≥10 Years	497	78.9
Sex	Male	305	48.4
	Female	325	51.6
Ethnicity	Oromo	409	64.9
	Amara	173	27.5
	Tigre	39	6.2
	Others^a^	9	1.4
Religion	Protestant	261	41.4
	Orthodox	251	39.8
	Muslim	109	17.3
	Others^b^	9	1.4
Grade attended	2-4 grade	222	35.2
	5-8 grade	408	64.8
Parental Respondents	Father/male guardians	130	20.6
	Mother/female guardians	500	79.4
Parental current marital status	Married	443	70.3
	Separated/ Divorced	187	29.7
Parental educational status	Not attended formal education	173	27.5
	Attended formal education	457	72.5
Parental occupational status	Unemployed	25	3.9
	Run own business	100	15.9
	Employed	505	80.2
Family size	Less than five	219	34.7
	Five and above	411	65.3
Average family monthly income	Less than 1000ETB	164	26.0
	1000-2000ETB	224	35.6
	More than 2000ETB	242	38.4
Regularly attend parent-teacher meeting	Yes	437	69.4
	No	193	30.6
Talk about schooling with children	Yes	326	51.7
	No	304	48.3
Support the children during home work	Yes	365	57.9
	No	265	42.1
Follow children at school	Yes	466	74.0
	No	164	26.0

*ETB* Ethiopian Birr, ^a^Others -Gurage, Gumuz ^b^Others-Wakeffata, cultural

### Socio-demographic characteristics and schooling support of the parents

In our study, socio-demographic and nutritional data were obtained from the parents/guardians of the students. For this purpose 500 (79.4%) mothers/female guardians and 130 (20.6%) fathers/male guardians of the students were included as secondary respondents. Four hundred forty three (70.3%) of the parents were currently in marriage and 457 (72.5%) of them attended formal education. Majority of the parents, 505 (80.2%) were employed while 100 (15.8%) and 25 (4%) were running their own business and unemployed respectively. Parents earning monthly income of more than 2000 Ethiopian Birr (~USD 91) accounted for 242 (38.4%) of the respondent. Four hundred thirty seven (69.4%) of the parents reported that they regularly attend the school meeting (parent-teacher meeting). Slightly more than half of the parents, 326 (51.7%), responded that they always discuss about their children schooling while still more proportion 365 (57.9%) of the parents support their children during home work. Majority of the parents 466 (74.0%) reported that they follow their children at school (Table 1).

### Anthropometric assessment

Anthropometric measurements; height and weight were taken and the nutritional indices, weight-for-age, height-for-age and weight-for-height were derived to assess the nutritional status of students. Mean height and weight of the study participants were 146.2 cm (SD ± 12.6 cm) and 38.3 Kg (SD ± 8.0 Kg) respectively (Table 2). Nutritional indices that are derived from anthropometric data indicated that prevalence of stunting, underweight and wasting in the study participants were 20.6%, 14.3% and 12.7% respectively.

**Table 2:** Anthropometric assessment of students by gender at primary school of Hawa Gelan District, Oromia Region, Southwest Ethiopia, 2016, (*N* = 630)

Variables	Male	Female	P-Value
Age in years	12.1 ± 2	12.3 ± 2	0.681
Weight in Kg	36.12 ± 6.92	34.38 ± 9.0	0.057
Height in cm	146.4 ± 12.3	146.2 ± 12.6	0.224
BMI in Kg/m^2^	16.9 ± 1.6	16.1 ± 1.8	0.052
Weight-for-age (WAZ)	−2.3 ± 1.2	−2.2 ± 1.3	<0.001
Height-for-age (HAZ)	−1.6 ± 1.0	−1.5 ± 1.0	0.043
Weight-for-height (WHZ)	−1.4 ± 1.1	−1.4 ± 1.2	0.082

*WAZ* Weight for Age Z-score, *HAZ* Height for Age Z-score, *WHZ* Weight for height Z-score

### Correlation of undernutrition and academic performance

Based on the cut-off point mark of 50%, 484 (76.8%) of the students achieved good score. Pearson’s correlation analysis was conducted to assess presence of correlation between academic performance and undernutrition among students. The result of correlation analysis indicated that stunting (HAZ) (*r* = 0.222, *p* = 0.04) and underweight (WAZ) (*r* = 0.214, *p* = 0.034) were statistically associated with academic performance of the students. However, wasting (WHZ) (*r* = 0.085, *p* = 0.135) was failed to show a significant correlation with students’ academic performance (Table 3).

**Table 3:** Pearson correlation between nutritional indicators and educational performance at primary schools of Hawa Gelan woreda, Oromia Region, South west Ethiopia, 2016, (*n* = 630)

Nutritional indicator	Average score	
	r	*p*-value**
WAZ	0.222	0.040*
HAZ	0.214	0.034*
WHZ	0.085	0.135*

*WAZ* Weight for Age Z-score, *HAZ* Height for Age Z-score, *WHZ* Weight for height Z-score

*Presence of statistically significant linear association

**Independent sample *t*-test

### Predictors of academic performance; the logistic regression analysis result

Multivariate logistic regression analysis indicated that stunting and underweight were significantly associated with students’ academic performance. Stunted students were less likely to achieve good academic performance as compared to students having no stunting [AOR 0.68; 95% CI (0.15, 0.85)]. Underweight students were less likely to achieve good academic performance as compared to students having normal weight [AOR 0.48; 95% CI (0.14, 0.84)]. Socio demographic factors including sex, grade attended, parental socio economic status and parental involvement in their children’s schooling were also found to be independently associated with academic performance. Female students were more likely to achieve good educational performance than their male counterpart [AOR 1.48; 95% CI (1.16, 3.82)]. Students attending above grade 4 are more likely to achieve good academic performance as compared to those students attending lower grade [AOR 2.12; 95% CI (1.98, 4.87)]. Students with educated parents were more likely to achieve good academic performance as compared to those students with uneducated parents [AOR 2.18; 95% CI (1.43, 4.72)]. Students from households with monthly income of more than 2000ETB were 2.85 times more likely to achieve good academic performance as compared to students from households with monthly income of less than 1000ETB [AOR 2.85; 95% CI (2.01, 5.21)]. Parental support during homework increases the likelihood of academic performance; students who have no parental support were less likely to achieve good academic performance as compared to those who have parental support [AOR 0.57; 95% CI (0.19, 0.98)] (Table 4).

**Table 4:** Factors associated with educational performance at primary schools of Hawa Gelan District, Oromia Region, Southwest Ethiopia, 2016, (*N* = 630)

Explanatory Variables	Educational performance		COR 95% CI	AOR 95% CI
	Good (≥50%) (76.8) 484	Poor (<50%) (23.2) 146		
Sex of the student 484				
Male	246	59	1.00	1.00
Female	238	87	1.52 (1.13,4.47)	1.48 (1.16, 3.82)*
Grade attended				
Grade 2-4	190	32	1.00	1.00
Above Grade 4	294	114	2.30 (1.72, 5.36)	2.12 (1.98, 4.87)*
Current parental marital status				
Married	333	110	1.00	
Divorced/widowed	151	36	0.72 (0.58, 0.94)	0.84 (0.69, 1.20)
Parental family size				
Less than five	167	52	1.00	1.00
Five and above	317	94	0.95 (0.69,2.30)	0.86 (0.67,1.98)
Parental education				
No formal education	148	25	1.00	1.00
Attend formal education	336	121	2.13 (1.82, 5.75)	2.18 (1.43, 4.72)*
Household monthly income				
Less 1000ETB	145	19	1.00	1.00
1000-2000ETB	165	59	2.73 (1.82, 4.94)	2.51 (2.12, 5.18)*
More than 2000ETB	174	68	2.98 (2.13, 6.11)	2.85 (2.01, 5.21)*
Parental support during homework				
Yes	267	98	1.00	1.00
No	217	48	0.60 (0.23, 0.94)	0.57 (0.19, 0.98)*
Parental follow up at school				
Yes	352	114	1.00	1.00
No	132	32	0.74 (0.39, 0.92)	0.72 (0.24, 1.36)
Participant stunted				
No	378	122	1.00	1.00
Yes	106	24	0.70 (.21, 0.94)	0.68 (0.15,0.85)*
Participant underweight				
No	408	132	1.00	1.00
Yes	76	14	0.56 (0.17, 0.82)	0.48 (0.14, 0.84)*
Participant wasted				
No	422	128	1.00	1.00
Yes	62	18	0.95 (0.53, 2.86)	0.86 (0.46, 2.23)

*indicates statistically significant association

## Discussion

The findings of current study showed that the average score for overall annual subjects of the students was found to be 67.2% with (SD ± 15.4%). Based on a cut-off point of 50% marks 146 (23.2%) of the students achieved poor academic performance. This study revealed lower underachievement when compared with a study conducted in Uganda in 2013 which reported 68.4% of the primary school students were underachievers [18]. This lower under achievement in the study area may be attributed to the better course delivery system and well structured curriculum.

Our study revealed high prevalence of undernutrition among students attending primary schools; 20.6% stunting, 14.2% underweight and 12.7% wasting. This findings are slightly lower than the finding of a study conducted in the Plantation Sector in Nuwara Eliya Educational Zone in which prevalence of stunting was 32.2%, underweight 50.4% and thinness 33.7% were reported [19]. This result also indicated lower prevalence of undernutrition than the study conducted in northern part of Ethiopia where prevalence of stunting, underweight and thinness were found to be 30.7%, 59.7% and 37.2% respectively [20]. However, the finding of current study indicated higher prevalence of undernutrition than that study conducted in India in which 20% had undernutrition; 7% stunting and 34% thinness [21]. The main reason for the observed differences in the present study could be due to poor socio-economic status of the study participants although variations in sex and the study period could contribute to the differences.

The current study has also established a positive correlation between two indicators of undernutrition and academic performance of students. In our study 218 (34.6%) of the students have any form of undernutrition that affects their educational achievements *P* < 0.001. Similar to this study, the most recent report of Save the Children stated that adults who were malnourished during childhood period earned twenty percent less in academic performance, on average, than those who were not [22]. Several other studies have found similar positive correlation of undernutrition and students’ academic performance [23–25].

According to the present study underweight (WAZ) and stunting (HAZ) have statistically significant association with lower academic performance as compared with children having normal weight (*P* value <0.05). A study conducted in Uganda reported similar findings that all nutritional indicators (HAZ and WAZ) had significant positive associations with learning achievement among children [26]. The finding of our study is also consistence with the study conducted in Nuwara Eliya in which weight for height and weight-for-age Z-scores showed significant positive association with overall subject average marks [19]. Similar finding was also reported from Ethiopia that indicated good HAZ score was significantly associated with higher academic scores [24]. The finding of this study is also consistence with the finding of a study conducted in Sri Lanka which showed that higher HAZ score is associated with better academic achievement [19]. These similar findings may be attributed to that height-for-age reflects the accumulation of nutritional deprivation throughout the years, which may consequently affect educational achievement of children.

In present study there was also observed differences in educational achievements between both sexes. Educational performance of female students was higher than that of male counterpart. This is in agreement with the study of Hall et al., 2001, in Vietnam and a study conducted in Ghana which reported that boys had a significantly lower test score than girls [27, 28]. However, our finding was inconsistence with the finding of study conducted in Nuwara Eliya which revealed that educational performance of female students was significantly higher than males except in Mathematics [19]. The possible explanations for male students under achievement may be boys spend most of their time on either working as helpers for their parents or playing outside with their friends and they tend to have a lower time to study. In contrast, girls spend more time in their homes and have the opportunity to study more than boys.

Multivariate logistic regression analysis in our study showed that there were a significant association between parental educational status, parental support during homework and household monthly income and students’ academic achievement. The findings of this study are consistence with the study conducted in Ghana where parental education and parental involvement were found as the main determinants of academic performance [28]. However, our finding is inconsistence with the study of Sarma et al, 2013 in Nuwara Eliya where the educational performance was significantly associated with types of schools [19]. The possible reasons for observed deference may be, as parents involved in their children schooling, they may help the students to spend most of their time on studying. Those educated parents may support the students to understand the courses they received.

This study had the following limitation. The first limitation was the usual drawback of the cross sectional design that cannot establish casual relationship between variables. There were imited number of similar published paper in the country that put challenges to compare our findings with. This study used only anthropomorphic measurements and did not assess the micronutrient status of study participants. Furthermore, there may have been differences in the evaluation system for students’ academic performance among the study schools. However, the sample size is large so that the finding could be generalized to students attending primary schools.

## Conclusion

Undernutrition (stunting and underweight) among children attending primary school is found to be correlated with academic performance. Parental involvement in schooling, parental academic and economic status was significantly associated with academic performance. Nutritional interventions should be undertaken to improve academic performance of primary school students. Parental involvement in their children schooling must be encouraged to improve their children’s academic ambitions. Further researches with better design should be conducted to identify correlation of undernutrition and academic performance.

## Abbreviations

ABH: Alliance for Better Health
CI: Confidence Interval
EFA: Education for All
ETB: Ethiopian Birr
HAZ: Height for Age Z-score
IRB: Institutional Review Board
SD: Standard Deviation
SPSS: Statistical Package for Social Science
WAZ: Weight for Age Z-score
WHO: World Health Organization
